# Trait Anxiety Mediates Impulsivity and Suicidal Ideation in Depression During COVID-19 Pandemic

**DOI:** 10.3389/fpsyt.2022.892442

**Published:** 2022-07-07

**Authors:** Xinyu Cheng, Yi Zhang, Di Zhao, Ti-Fei Yuan, Jianyin Qiu

**Affiliations:** ^1^Shanghai Key Laboratory of Psychotic Disorders, Shanghai Mental Health Center, Shanghai Jiao Tong University School of Medicine, Shanghai, China; ^2^Co-innovation Center of Neuroregeneration, Nantong University, Nantong, China; ^3^Shanghai Mental Health Center, Shanghai Jiao Tong University School of Medicine, Shanghai, China

**Keywords:** suicidal ideation, major depressive disorder, impulsivity, anxiety, COVID-19 pandemic

## Abstract

Suicidality in patients with major depressive disorder (MDD) has been an urgent affair during the COVID-19 pandemic. It is well-established that impulsivity and trait anxiety are two risk factors for suicidal ideation. However, literature is still insufficient on the relationships among impulsivity, (state/trait) anxiety and suicidal ideation in individuals with MDD. The present study aims to explore the relationships of these three variables in MDD patients during the COVID-19 pandemic through three scales, including Barrett Impulsivity Scale (BIS), State-Trait Anxiety Scale (STAI) and Self-rating Idea of Suicide Scale (SIOSS). Sixty-three MDD patients (low SIOSS group and high SIOSS group, which were split by the mean score of SIOSS) and twenty-seven well-matched healthy controls were analyzed. Our results showed that the high SIOSS group had higher trait anxiety (*p* < 0.001, 95% CI = [−19.29, −5.02]) but there was no difference in state anxiety (*p* = 0.171, 95% CI = [−10.60, 1.25]), compared with the low SIOSS group. And the correlation between impulsivity and suicidal ideation was significant in MDD patients (*r* = 0.389, *p* = 0.002), yet it was not significant in healthy controls (*r* = 0.285, *p* = 0.167). Further, mediation analysis showed that trait anxiety significantly mediate impulsivity and suicidal ideation in patients with depression (total effect: β = 0.304, *p* = 0.002, 95% CI = [0.120, 0.489]; direct effect: β = 0.154, *p* = 0.076, 95% CI = [−0.169, 0.325]), indicating impulsivity influenced suicidal ideation through trait anxiety in MDD patients. In conclusion, our results suggested that trait anxiety might mediate the association of impulsivity and suicidal ideation in MDD patients. Clinicians may use symptoms of trait anxiety and impulsivity for screening when actively evaluating suicidal ideation in MDD patients, especially in the setting of COVID-19 pandemic.

## Introduction

Mental problems after COVID-19 outbreak arouse public concern (1–3). Latest statistics show that the rise of major depressive disorder (MDD) is 27.6% during the pandemic (4). Patients with MDD may experience worsening symptoms and their prevalence of suicidal ideation during the pandemic is 66.4% (5–8). Hence, suicidal ideation is a critical issue in patients with MDD during the pandemic. It is urgent to explore factors associated with suicidal ideation in MDD.

Impulsivity is a prominent factor of suicidality and may lead to an increased risk of suicidality (9–12). It refers to quick response and poor planning before considering consequences (13). Impulsive individuals may have a poor ability to get rid of suicidal thoughts or control their behaviors, thus causing tragedies. Wang et al. have found that MDD patients with higher impulsivity tend to have more suicidal ideation (14). Moreover, subcortical atrophy (e.g., globus pallidus) alterations have been revealed in patients with MDD and were severity of suicidal ideation and impulsivity dependent (15).

Although a majority of research has identified associations between impulsivity and suicidal ideation in individuals with MDD, the effect of anxiety is underestimated. Anxiety is a widely-explored risky predictor of suicidality (16–19). Recent research has demonstrated the correlation between anxiety symptoms and suicidality in MDD patients (20). This evidence displays the potential impact of anxiety on suicidal risk in MDD patients. Furthermore, anxiety symptoms commonly occur with MDD (21, 22) and would be intensified by COVID-19 pandemic (1, 5). Thereby, the influence of anxiety on suicidality should not be overlooked, especially during the pandemic. Patients with MDD are likely to have a higher level of anxiety, and subsequently, a higher risk of suicidality. Due to the negative impact, prevalence and severity of anxiety in the pandemic, great attention should be paid to anxiety in MDD patients.

The literature on the relationship of impulsivity, anxiety, and suicidal ideation is still lacking. A broad of evidence has verified the connection between impulsivity and anxiety, but their relationship has not reached a consensus yet (23–27). Notably, some studies have demonstrated the contribution of impulsivity to internalizing psychopathology [e.g., depression and anxiety; (28, 29)]. That impulsivity predicts anxiety severity has been found in both children (29) and adults (24) who are with internalizing or externalizing symptoms. Specifically, impulsivity would engage individuals in a highly emotional state in a stressful setting, causing anxiety symptoms (24). Hence, it could be inferred that impulsivity would lead to a higher level of anxiety during the pandemic. Trait anxiety is characterized as a general level of stress and anxiety, while state anxiety refers to a state of emotional stress and anxiety that responds to a fearful or dangerous situation (18, 30, 31). Previous research has suggested trait anxiety would amplify impulsivity, resulting in severer consequences (23). Extensive studies have indicated trait anxiety might be a more important predictor of suicide risk than state anxiety (18, 32). Based on the mentioned studies, trait anxiety may mediate the association between impulsivity and suicidal ideation.

Studies regarding the influence of impulsivity and trait anxiety on suicide ideation in MDD patients are still scarce. Our study aimed to investigate the relationship of impulsivity and anxiety on suicide ideation in an outpatient sample with MDD in the context of COVID-19 pandemic. We hypothesized that MDD individuals with higher suicidal ideation would exhibit increased impulsivity and trait anxiety. We also hypothesized that impulsivity was associated with suicidal ideation, which was mediated by trait anxiety in patients with MDD.

## Materials and Methods

### Study Design and Sample Size

This study was designed as a case-control study, expecting to recruit a depressed group and a healthy control group. The sample sizes of patients and healthy subjects were calculated by using two independent proportions in PASS 15. Since the depressive group needed to be divided into two subgroups (groups with low and high suicidal ideation) for stratified comparison, the ratio of the sample size of healthy subjects to the sample size of patients should be 0.5. The exposure rates of patients (P1) and of healthy subjects (P2) were approximately estimated as 0.84 and 0.5 separately, according to a previous study related to suicidality and impulsivity (33). Power and alpha were set as 0.9 and 0.05, respectively. The results showed that the sample sizes of the depressive group should be 55 and the healthy group should be 28.

### Subjects

**FIGURE 1:**
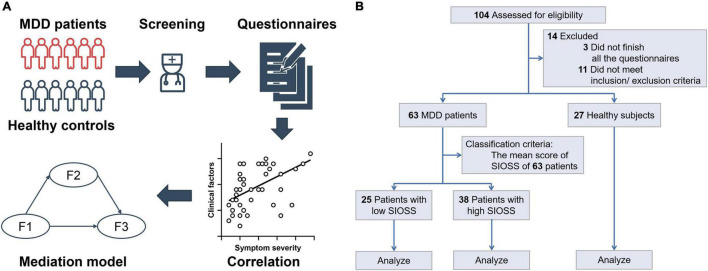
The procedure of the study. **(A)** Study procedure; **(B)** The flow chart of sample size.

We recruited 77 patients and 27 healthy subjects from April to September 2021, which was during the period of the COVID-19 pandemic. All patients should meet the criteria for depressive episodes according to the Mini-International Neuropsychiatric Interview (MINI) and the Hamilton Depression Scale (score ≥ 17). Exclusion criteria included the current or past history of psychotic disorders, bipolar disorder, history of alcohol/substance abuse, active medical or neurological problems, and other psychiatric diseases. After screening, 63 patients diagnosed with MDD (28.54 ± 6.75 years) and 27 well-matched healthy subjects (29.22 ± 10.87 years) were analyzed (see more details in Figure 1B). Part of the patients received or had been receiving drug treatment. This study was approved by the ethics committee of Shanghai Mental Health Center (ethics committee approval number: CRC2017YB01) and complied with the Declaration of Helsinki. The procedure of this study is shown in Figure 1A.

### Questionnaires

The Hamilton Depression Scale (HAMD) (34) is a widely-used assessment for depressive severity (14, 35, 36), including 17 items on a 5-point scale (0 = “none”, 4 = “extremely severe”). Higher scores represent severer depression symptoms. The scale is examined by a trained rater by conversation and observation.

The Hamilton Anxiety Scale (HAMA) (37) is a widely-used assessment for anxious severity (35, 36, 38), including 14 items on a 5-point scale (0 = “none”, 4 = “extremely severe”). Higher scores represent severer anxiety symptoms. The scale is examined by a trained rater by conversation and observation.

The Barrett Impulsivity Scale (BIS) (39) is a self-rating scale used to evaluate impulsivity, containing 30 items on a 5-point scale (1 = “never”, 5 = “always”). It consists of three dimensions: motor impulsivity, attentional impulsivity, and non-planning impulsivity, with a total score varying between 30 and 150. High scores represent hyperactivity, inattention, and lack of planning (i.e., higher impulsivity). Cronbach’s alpha for BIS was 0.652 in the present study.

The 26-item Self-rating Idea of Suicide Scale (SIOSS) (40) is a self-rating scale used to evaluate suicidal ideation. The scale includes four factors: despair, sleep, optimism, and concealment. Participants should choose “yes” or “no” as the answer. If the concealment factor is ≥4, the result is invalid and should be excluded. High scores represent stronger suicidal ideation. Cronbach’s alpha for SIOSS was 0.652 in the present study.

The 40-item State-Trait Anxiety Scale (STAI) (31) is a self-rating scale used to evaluate state and trait anxiety on a 4-point scale (1 = “not at all”, 4 = “very much so”). The scale has two subscales: (1) State Anxiety Inventory (STAI-S) measures state anxiety, including items 1–20. State anxiety describes momentary and unpleasant emotions such as tension, fear, and worry. (2) Trait Anxiety Inventory (STAI-T) measures trait anxiety, including items 21–40. Trait anxiety describes a personality or a relatively stable tendency to feel anxious. Cronbach’s alpha for STAI was 0.638 in the present study.

### Statistical Analysis

Patients were divided into two subgroups (low/high SIOSS group) according to the mean of SIOSS of all patients, in order to distinguish the features of MDD patients with low and high suicidal ideation. One-way ANOVA analyses were used by IBM SPSS Statistics 24 (SPSS) on the demographic and clinical variables to compare the low SIOSS group, high SIOSS group, and healthy group. When significance was found in one-way ANOVA analysis, Bonferroni correction was then used in *post hoc* analysis. Pearson correlation analyses were performed by GraphPad Prism 8 to examine the relationships of clinical factors (suicidal ideation/impulsivity/trait anxiety) and symptom severity (HAMD/HAMA). To verify the relationships of suicidal ideation, impulsivity, and trait anxiety in depression, pairwise relationships of these three variables were first examined by Pearson correlation analyses. A mediation analysis was then performed by employing SPSS PROCESS Model 4. Similar analyses were also conducted in the healthy group. All statistical tests were two-tailed and the significance threshold was *p* < 0.05.

## Results

### Demographic and Clinical Characteristics

Sixty-three MDD patients were divided into two subgroups by the mean score of SIOSS. Of all the patients, 25 (39.7%) were divided into the low SIOSS group (SIOSS < 15) and 38 (60.3%) were divided into the high SIOSS group (SIOSS ≥ 15). The two subgroups and healthy group did not differ on demographic characteristics, except sleep quality (see Table 1). Clinically, both subgroups showed more severe depression symptoms (low SIOSS group: *p* < 0.001, 95% CI = [17.60, 23.71]; high SIOSS group: *p* < 0.001, 95% CI = [19.61, 25.15]) and anxiety symptoms (low SIOSS group: *p* < 0.001, 95% CI = [16.64, 25.14]; high SIOSS group: *p* < 0.001, 95% CI = [19.33, 27.03]) t compared with healthy group, while two MDD subgroups showed no significant difference in depression severity (*p* = 0.422) and anxiety severity (*p* = 0.479).

**TABLE 1:** Demographic and clinical characteristics of patients with low suicidal ideation, patients with high suicidal ideation, and healthy subjects.

		Low SIOSS group			High SIOSS group			Healthy group			Adjusted *p*	η ^2^	*Post hoc*
		*n*	Mean	SD	*n*	Mean	SD	*n*	Mean	SD			
Demographic information	Gender (Male: Female)	10:15			11:27			9:18			0.669	0.009	–
	Age	25	30.08	7.19	38	27.53	6.34	27	29.22	10.87	0.451	0.018	–
	Education (years)	25	16.20	3.49	38	14.89	2.57	27	15.89	2.04	0.138	0.044	–
	AUDIT	25	2.76	6.04	38	3.21	5.84	27	0.67	1.94	0.127	0.046	–
	FTND	25	0.56	1.56	38	0.37	1.40	27	0.00	0.00	0.246	0.032	–
	PSQI	25	10.00	4.07	38	10.89	3.19	27	4.48	2.16	<0.001	0.441	HC < LS, HS
Scales	HAMD	25	21.88	4.82	38	23.61	5.54	27	1.22	1.67	<0.001	0.835	HC < LS, HS
	HAMA	25	22.00	7.58	38	24.29	7.30	27	1.11	1.60	<0.001	0.734	HC < LS, HS
	BIS no plan	25	29.16	7.09	38	33.08	6.51	27	22.15	5.54	<0.001	0.346	HC < LS, HS
	BIS motor	25	26.44	6.40	38	29.34	5.59	27	21.37	6.07	<0.001	0.245	HC < LS, HS
	BIS attention	25	27.08	5.50	38	27.63	5.19	27	22.63	4.67	0.001	0.160	HC < LS, HS
	BIS total	25	27.56	4.98	38	30.02	4.75	27	22.05	4.50	<0.001	0.342	HC < LS, HS
	STAI-S	25	58.40	10.72	38	63.08	9.92	27	33.41	7.15	<0.001	0.659	HC < LS, HS
	STAI-T	25	56.40	13.41	38	68.55	9.51	27	36.85	11.67	<0.001	0.586	HC < LS < HS
	SIOSS despair	25	6.44	2.16	38	10.66	1.17	25	2.32	2.81	<0.001	0.751	HC < LS < HS
	SIOSS sleep	25	2.64	1.38	38	3.26	0.92	25	0.88	1.17	<0.001	0.444	HC < LS, HS
	SIOSS optimism	25	2.00	1.16	38	3.87	0.70	25	0.36	0.91	<0.001	0.730	HC < LS < HS
	SIOSS concealment	25	0.68	0.90	38	0.76	0.82	25	0.72	0.79	0.927	0.002	–
	SIOSS total	25	11.08	2.60	38	17.79	1.58	25	3.56	3.99	<0.001	0.828	HC < LS < HS

*AUDIT, Alcohol Use Disorders Identification Test; FTND, Fagerstrom Test for nicotine dependence; PSQI, Pittsburgh Sleep Quality Index; HAMD, Hamilton Depression Scale; HAMA, Hamilton Anxiety Scale; BIS, Barrett Impulsivity Scale; STAI, State-Trait Anxiety Scale; SIOSS, Self-rating Idea of Suicide Scale; HC, healthy group; LS, low SIOSS group; HS, high SIOSS group.*

### Impulsivity

**FIGURE 2:**
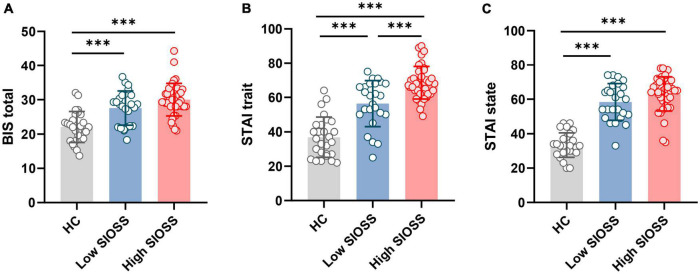
Comparisons of healthy controls, patients with low suicidal ideation, and patients with high suicidal ideation on clinical factors. **(A)** Comparisons of impulsivity between three groups; **(B)** Comparisons of trait anxiety between three groups; **(C)** Comparisons of state anxiety between three groups. ***p < 0.001.

One-way ANOVAs were performed on impulsivity. Results showed that the low SIOSS group, high SIOSS group, and healthy group differed on the total score of BIS [F(2, 87) = 22.56, *p* < 0.001, η^2^ = 0.342]. *Post hoc* analyses revealed that the two MDD subgroups showed no difference in the score of BIS (*p* = 0.142, 95% CI = [−5.44, 0.52]). However, the total scores of BIS of low SIOSS group (*p* < 0.001, 95% CI = [2.30, 8.72]) and high SIOSS group were both higher than the healthy group (*p* < 0.001, 95% CI = [5.05, 10.88]; Figure 2A). The results indicated that patients had higher impulsivity than healthy subjects, but impulsivity did not increase despite stronger suicidal ideation.

### State and Trait Anxiety

The results of one-way ANOVAs revealed significant difference among three groups on trait anxiety [F(2, 87) = 84.15, *p* < 0.001, η^2^ = 0.566] and state anxiety [F(2, 87) = 61.66, *p* < 0.001, η^2^ = 0.659]. *Post hoc* analysis revealed that both two MDD subgroups showed higher trait anxiety (low SIOSS group: *p* < 0.001, 95% CI = [11.86, 27.24]; high SIOSS group: *p* < 0.001, 95% CI = [24.73, 38.67]) and higher state anxiety (low SIOSS group: *p* < 0.001, 95% CI = [18.61, 31.38]; high SIOSS group: *p* < 0.001, 95% CI = [23.88, 35.46]) than healthy group (Figures 2B,C). However, low SIOSS group and high SIOSS group differed on trait anxiety (*p* < 0.001, 95% CI = [−19.29, −5.02]) but not on state anxiety (*p* = 0.171, 95% CI = [−10.60, 1.25]). These results suggested that severe state and trait anxiety were presented in MDD patients, and only trait anxiety was severer in MDD patients with higher suicidal ideation.

### Correlations Between Clinical Indexes and Symptom Severity

**FIGURE 3:**
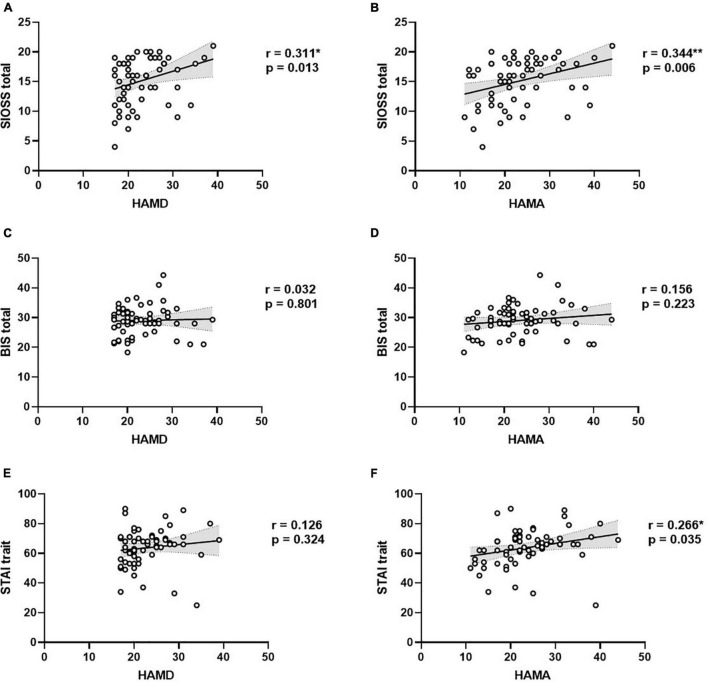
Correlations between clinical indexes and symptom severity in MDD. **(A)** Correlation between suicidal ideation and depression severity; **(B)** Correlation between suicidal ideation and anxiety severity; **(C)** Correlation between impulsivity and depression severity; **(D)** Correlation between impulsivity and anxiety severity; **(E)** Correlation between trait anxiety and depression severity; **(F)** Correlation between trait anxiety and anxiety severity.

We used Pearson correlation analyses to examine the relationships between symptom severity and clinical factors (see Figures 3A–F). The results showed that suicidal ideation was associated with HAMD (*r* = 0.311, *p* = 0.013) and HAMA (*r* = 0.344, *p* = 0.006; Figures 3A,B). These results demonstrated that MDD patients with severer depression and anxiety symptoms tended to have stronger suicidal ideation. Additionally, trait anxiety was associated with HAMA (*r* = 0.266, *p* = 0.035; Figure 3F). Correlation analyses demonstrated that MDD patients with severer anxiety symptoms would have a higher level of trait anxiety.

### Impulsivity Influences Suicidal Ideation Through Trait Anxiety

**FIGURE 4:**
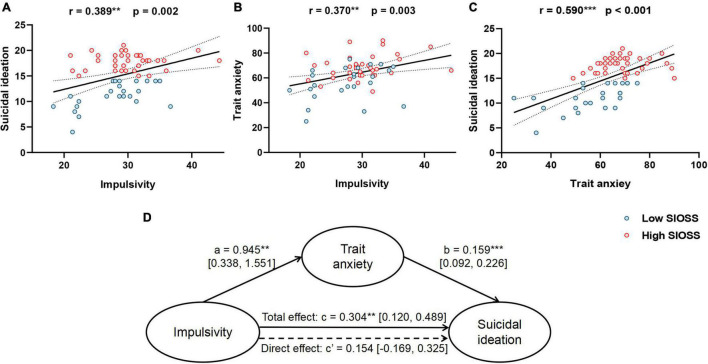
Correlations and mediation model. **(A)** Correlation between impulsivity and suicidal ideation; **(B)** Correlation between impulsivity and trait anxiety; **(C)** Correlation between trait anxiety and suicidal ideation; **(D)** Mediation model. **p < 0.01, ***p < 0.001.

Pearson correlation analyses were performed to test the pairwise relationships among impulsivity, trait anxiety, and suicidal ideation in patients. The results showed that impulsivity was associated with suicidal ideation (*r* = 0.389, *p* = 0.002) and trait anxiety (*r* = 0.370, *p* = 0.003; Figures 4A,B). And trait anxiety was significantly correlated with suicidal ideation as well (*r* = 0.590, *p* < 0.001; Figure 4C). Additionally, the same analysis was also conducted on impulsivity and state anxiety and revealed no significant correlation (*r* = 0.134, *p* = 0.294). The results suggested that impulsivity only correlated with trait anxiety.

Given these pairwise relationships, a mediation analysis was performed to test whether trait anxiety mediated the relationship between impulsivity and suicidal ideation in MDD. The total effect of impulsivity on suicidal ideation was significant (β = 0.304, *p* = 0.002, 95% CI = [0.120, 0.489], Figure 4D). However, the direct effect of impulsivity on suicidal ideation was not significant (β = 0.154, *p* = 0.076, 95% CI = [−0.169, 0.325]). These results suggested trait anxiety might play a fully mediating role between impulsivity and suicidal ideation in depression. The results supported that impulsivity might lead to suicidal ideation through trait anxiety instead of directly influencing suicidal ideation in MDD patients.

As for healthy subjects, same analyses were used to examine the relationship of impulsivity, trait anxiety and suicidal ideation. Pearson correlation analyses showed trait anxiety was positively correlated with suicidal ideation (*r* = 0.442, *p* = 0.027) while impulsivity have no correlation with trait anxiety (*r* = 0.020, *p* = 0.921) and suicidal ideation (*r* = 0.285, *p* = 0.167). These findings indicated that impulsivity has no correlation with trait anxiety and suicidal ideation in healthy subjects.

## Discussion

The results demonstrated that MDD patients with stronger suicidal ideation have higher impulsivity and trait anxiety, which was consistent with previous studies (20, 26, 41). Furthermore, the results showed trait anxiety played a fully mediating role between impulsivity and suicidal ideation in depression. The associations found among impulsivity, trait anxiety and suicidal ideation contribute to the current literature, and indicate that those individuals with MDD who have higher level of impulsivity and trait anxiety may be at risk for suicidal ideation.

The findings revealed that impulsivity had an impact on suicidal ideation through trait anxiety rather than state anxiety in MDD. The relationship of impulsivity and trait anxiety is consistent with the previous studies (24, 29). As extensive studies have revealed, impulsive individuals would have deficits in active coping (42) and emotion regulation (43, 44). Based on the data in the present study, it seems reasonable to assume that MDD patients with higher impulsivity would lack active coping to deal with the distress and negative emotions caused by COVID-19 pandemic, resulting in frequent, aggravated and long-lasting negative emotions [i.e., trait anxiety; (30, 45)]. Our findings also accord with previous studies and show that due to higher trait anxiety, individuals would then have stronger suicidal ideation (18, 32). Thereby, the mediating role of trait anxiety is observed in the association of impulsivity and suicidal ideation. However, these findings do not contradict accumulating evidence that impulsivity is an outcome or a moderator of anxiety (26, 27). Yu et al. reveal the impact of anxiety on impulsivity in college students (27), while Schaefer et al. demonstrate impulsivity moderates the association between anxiety and suicidal ideation in undergraduate students (26). Combined with the existing evidence, the relationship between impulsivity and anxiety may have its complexity. Future research could explore the influence of interactions between impulsivity and anxiety on suicidal risk in different situations or clinical populations.

Moreover, we found correlations between depression/anxiety severity and suicidal ideation, which are consistent with previous studies (14, 38). The results indicate that MDD patients with severe depression and anxiety symptoms are also at high risk of suicidality. Therefore, those patients with severe depression and anxiety symptoms need great concern. Additionally, the results showed that impulsivity was not correlated with depression severity (i.e., the score of HAMD) and anxiety severity (i.e., the score of HAMA), which is contradictory to the finding of Johnson et al. (24). One possibility is the heterogeneity of measurements. Specifically, the Mood and Anxiety Symptoms Questionnaire (MASQ), used by Johnson et al., assesses core depression/anxiety symptoms from affectivity and somatic arousal (46). Yet, HAMA and HAMD measure some medication side effects or border symptoms (e.g., sleep, weight and sexual problems, and hypochondriasis) besides core symptoms (34, 37, 47). Our study corroborates the previous findings (24) by showing trait anxiety was correlated with impulsivity. The contradiction may indicate that impulsivity is mainly connected with the affective aspect of symptoms. It is essential for future studies further explore the relationship of impulsivity and negative effects or affective experiences. Alternatively, MASQ is a self-reported scale, while HAMA and HAMD are rated by trained raters. The way of rating may also cause inconsistent findings.

The findings suggest several clinical implications. Firstly, extra measurement tools (e.g., BIS and STAI) may assist the examination of suicidal risk in MDD patients. Furthermore, clinicians should put more emphasis on MDD patients with higher trait anxiety and impulsivity, and could consider interventions centered on anxiety and impulsivity to reduce suicidal risk. According to several clinical studies, Cognitive Anxiety Sensitivity Treatment (CAST) and Dialectical Behavior Therapy (DBT) target anxiety symptoms and suicidality, and have good efficacy in reducing suicidal ideation (48, 49). Besides, a brief online intervention is also proved to be efficient to reduce emotion-related impulsivity, which includes emotion recognization, self-calming techniques and pre-plan coping strategies (50). Retention and engagement of interventions would be challenging (51), especially considering the high level of impulsivity in MDD patients in our study. These impulsive individuals have difficulties in focusing on the task and long-term goals, and therefore, it is necessary to highlight the long-term goals and consequences of imprudent behaviors during the intervention (51).

Several limitations need to be mentioned in the present study. First, the relatively small sample and different sample sizes of each group (low SIOSS group/high SIOSS group/healthy group) may result in possible bias. If more subjects had been recruited, we may have demonstrated the difference in impulsivity between MDD patients with low suicidal ideation and with high suicidal ideation. Second, the self-report scale could also cause bias due to the honesty, education, and statement of individuals. Future work could recruit a larger sample of patients and adopt behavioral measurements to verify the relationships among impulsivity, trait anxiety, and suicidal ideation. Third, this study does not include the history of suicidal behaviors of MDD patients. Besides suicidal thoughts, suicidal behaviors are also an important part of suicidality in MDD patients, which is also necessary for exploration in the future.

In conclusion, impulsivity and trait anxiety may be the risk factors for suicidal ideation in depression. Besides, trait anxiety plays a fully mediating role between impulsivity and suicidal ideation. These findings indicate impulsivity has an impact on suicidal ideation through trait anxiety in MDD. Additionally, during COVID-19 pandemic, attention should be paid to MDD patients with high anxiety symptoms and impulsivity to prevent and intervene in the potential suicidal thoughts and behaviors.

## Data Availability Statement

The raw data supporting the conclusions of this article will be made available by the authors, without undue reservation.

## Ethics Statement

The studies involving human participants were reviewed and approved by the Ethics Committee of Shanghai Mental Health Center. The patients/participants provided their written informed consent to participate in this study.

## Author Contributions

XC and YZ performed the study. XC, YZ, DZ, T-FY, and JQ designed the experiment, analyzed the results, wrote the manuscript, read, and approved the final version of the manuscript. All authors contributed to the article and approved the submitted version.

## Conflict of Interest

The authors declare that the research was conducted in the absence of any commercial or financial relationships that could be construed as a potential conflict of interest.

## Publisher’s Note

All claims expressed in this article are solely those of the authors and do not necessarily represent those of their affiliated organizations, or those of the publisher, the editors and the reviewers. Any product that may be evaluated in this article, or claim that may be made by its manufacturer, is not guaranteed or endorsed by the publisher.

## References

[1] Fancourt D Steptoe A Bu F. Trajectories of anxiety and depressive symptoms during enforced isolation due to COVID-19 in England: a longitudinal observational study. *Lancet Psychiatry.* (2021) 8:141–9. 10.1016/s2215-0366(20)30482-x 33308420 PMC7820109

[2] John A Pirkis J Gunnell D Appleby L Morrissey J. Trends in suicide during the covid-19 pandemic. *BMJ.* (2020) 371:m4352. 10.1136/bmj.m4352 33184048

[3] Kawohl W Nordt C. COVID-19, unemployment, and suicide. *Lancet Psychiatry.* (2020) 7:389–90. 10.1016/s2215-0366(20)30141-3 32353269 PMC7185950

[4] Santomauro DF Mantilla Herrera AM Shadid J Zheng P Ashbaugh C Pigott DM Global prevalence and burden of depressive and anxiety disorders in 204 countries and territories in 2020 due to the COVID-19 pandemic. *Lancet.* (2021) 398:1700–12. 10.1016/s0140-6736(21)02143-7 34634250 PMC8500697

[5] Fountoulakis KN Apostolidou MK Atsiova MB Filippidou AK Florou AK Gousiou DS Self-reported changes in anxiety, depression and suicidality during the COVID-19 lockdown in Greece. *J Affect Disord.* (2021) 279:624–9. 10.1016/j.jad.2020.10.061 33190113 PMC7605790

[6] Klomek AB. Suicide prevention during the COVID-19 outbreak. *Lancet Psychiatry.* (2020) 7:390. 10.1016/s2215-0366(20)30142-5 PMC7185940 32353271

[7] Mazza MG De Lorenzo R Conte C Poletti S Vai B Bollettini I Anxiety and depression in COVID-19 survivors: role of inflammatory and clinical predictors. *Brain Behav Immun.* (2020) 89:594–600. 10.1016/j.bbi.2020.07.037 32738287 PMC7390748

[8] Zhang L Cai H Bai W Zou SY Feng KX Li YC Prevalence of suicidality in clinically stable patients with major depressive disorder during the COVID-19 pandemic. *J Affect Disord.* (2022) 307:142–8. 10.1016/j.jad.2022.03.042 35337925 PMC8938301

[9] Gvion Y Levi-Belz Y Hadlaczky G Apter A. On the role of impulsivity and decision-making in suicidal behavior. *World J Psychiatry.* (2015) 5:255–9. 10.5498/wjp.v5.i3.255 26425440 PMC4582302

[10] Mann JJ Waternaux C Haas GL Malone KM. Toward a clinical model of suicidal behavior in psychiatric patients. *Am J Psychiatry.* (1999) 156:181–9. 10.1176/ajp.156.2.181 9989552

[11] Oliffe JL Rossnagel E Seidler ZE Kealy D Ogrodniczuk JS Rice SM. Men’s depression and suicide. *Curr Psychiatry Rep.* (2019) 21:103. 10.1007/s11920-019-1088-y 31522267

[12] Swann AC Dougherty DM Pazzaglia PJ Pham M Steinberg JL Moeller FG. Increased impulsivity associated with severity of suicide attempt history in patients with bipolar disorder. *Am J Psychiatry.* (2005) 162:1680–7. 10.1176/appi.ajp.162.9.1680 16135628

[13] Moeller FG Barratt ES Dougherty DM Schmitz JM Swann AC. Psychiatric aspects of impulsivity. *Am J Psychiatry.* (2001) 158:1783–93. 10.1176/appi.ajp.158.11.1783 11691682

[14] Wang YY Jiang NZ Cheung EF Sun HW Chan RC. Role of depression severity and impulsivity in the relationship between hopelessness and suicidal ideation in patients with major depressive disorder. *J Affect Disord.* (2015) 183:83–9. 10.1016/j.jad.2015.05.001 26001667

[15] Kim K Shin JH Myung W Fava M Mischoulon D Papakostas GI Deformities of the globus pallidus are associated with severity of suicidal ideation and impulsivity in patients with major depressive disorder. *Sci Rep.* (2019) 9:7462. 10.1038/s41598-019-43882-4 31097766 PMC6522489

[16] Casey SM Varela A Marriott JP Coleman CM Harlow BL. The influence of diagnosed mental health conditions and symptoms of depression and/or anxiety on suicide ideation, plan, and attempt among college students: findings from the healthy minds study, 2018-2019. *J Affect Disord.* (2022) 298:464–71. 10.1016/j.jad.2021.11.006 34774646

[17] Khan A Leventhal RM Khan S Brown WA. Suicide risk in patients with anxiety disorders: a meta-analysis of the FDA database. *J Affect Disord.* (2002) 68:183–90. 10.1016/s0165-0327(01)00354-8 12063146

[18] Ohring R Apter A Ratzoni G Weizman R Tyano S Plutchik R. State and trait anxiety in adolescent suicide attempters. *J Am Acad Child Adolesc Psychiatry.* (1996) 35:154–7. 10.1097/00004583-199602000-00007 8720624

[19] Wenzel A Beck AT. A cognitive model of suicidal behavior: theory and treatment. *Appl Prev Psychol.* (2008) 12:189–201. 10.1016/j.appsy.2008.05.001

[20] Saade YM Nicol G Lenze EJ Miller JP Yingling M Wetherell JL Comorbid anxiety in late-life depression: relationship with remission and suicidal ideation on venlafaxine treatment. *Depress Anxiety.* (2019) 36:1125–34. 10.1002/da.22964 31682328 PMC6891146

[21] Craske MG Stein MB. Anxiety. *Lancet.* (2016) 388:3048–59. 10.1016/s0140-6736(16)30381-6 27349358

[22] Gili M Toro MG Armengol S Garcia-Campayo J Castro A Roca M. Functional impairment in patients with major depressive disorder and comorbid anxiety disorder. *Can J Psychiatry.* (2013) 58:679–86. 10.1177/070674371305801205 24331287

[23] Beauchaine TP Zisner AR Sauder CL. Trait impulsivity and the externalizing spectrum. *Annu Rev Clin Psychol.* (2017) 13:343–68. 10.1146/annurev-clinpsy-021815-093253 28375718

[24] Johnson SL Porter PA Modavi K Dev AS Pearlstein JG Timpano KR. Emotion-related impulsivity predicts increased anxiety and depression during the COVID-19 pandemic. *J Affect Disord.* (2022) 301:289–99. 10.1016/j.jad.2022.01.037 35026359 PMC8747782

[25] Moustafa AA Tindle R Frydecka D Misiak B. Impulsivity and its relationship with anxiety, depression and stress. *Compr Psychiatry.* (2017) 74:173–9. 10.1016/j.comppsych.2017.01.013 28171742

[26] Schaefer KE Esposito-Smythers C Riskind JH. The role of impulsivity in the relationship between anxiety and suicidal ideation. *J Affect Disord.* (2012) 143:95–101. 10.1016/j.jad.2012.05.034 22925350

[27] Yu Y Yu Y Lin Y. Anxiety and depression aggravate impulsiveness: the mediating and moderating role of cognitive flexibility. *Psychol Health Med.* (2020) 25:25–36. 10.1080/13548506.2019.1601748 30947527

[28] Carver CS Johnson SL. Impulsive reactivity to emotion and vulnerability to psychopathology. *Am Psychol.* (2018) 73:1067–78. 10.1037/amp0000387 30525782 PMC6309622

[29] Cosi S Hernández-Martínez C Canals J Vigil-Colet A. Impulsivity and internalizing disorders in childhood. *Psychiatry Res.* (2011) 190:342–7. 10.1016/j.psychres.2011.05.036 21665292

[30] Knowles KA Olatunji BO. Specificity of trait anxiety in anxiety and depression: meta-analysis of the state-trait anxiety inventory. *Clin Psychol Rev.* (2020) 82:101928. 10.1016/j.cpr.2020.101928 33091745 PMC7680410

[31] Spielberger CD. *STAI Manual for the State-Trait Anxiety Inventory. Self-Evaluation Questionnaire.* Palo Alto, CA: Consulting Psychologists Press (1970). p. 1–24.

[32] Enãtescu I Craina M Gluhovschi A Giurgi-Oncu C Hogea L Nussbaum LA The role of personality dimensions and trait anxiety in increasing the likelihood of suicide ideation in women during the perinatal period. *J Psychosom Obstet Gynaecol.* (2021) 42:242–52. 10.1080/0167482x.2020.1734790 32116087

[33] Pang Y Tong Y Yuan X Wang X Yin Y Li L Impulsiveness and suicide attempts in rural areas. *Chin J Health Educ.* (2021) 37:525–30.

[34] Hamilton M. A rating scale for depression. *J Neurol Neurosurg Psychiatry.* (1960) 23:56–62. 14399272 10.1136/jnnp.23.1.56 PMC495331

[35] Liu Z Qiao D Xu Y Zhao W Yang Y Wen D The efficacy of computerized cognitive behavioral therapy for depressive and anxiety symptoms in patients with COVID-19: randomized controlled trial. *J Med Internet Res.* (2021) 23:e26883. 10.2196/26883 33900931 PMC8128049

[36] Richard-Devantoy S Ding Y Lepage M Turecki G Jollant F. Cognitive inhibition in depression and suicidal behavior: a neuroimaging study. *Psychol Med.* (2016) 46:933–44. 10.1017/s0033291715002421 26670261

[37] Hamilton M. The assessment of anxiety states by rating. *Br J Med Psychol.* (1959) 32:50–5. 10.1111/j.2044-8341.1959.tb00467.x 13638508

[38] Khansa W Haddad C Hallit R Akel M Obeid S Haddad G Interaction between anxiety and depression on suicidal ideation, quality of life, and work productivity impairment: results from a representative sample of the Lebanese population. *Perspect Psychiatr Care.* (2020) 56:270–9. 10.1111/ppc.12423 31321788

[39] Patton JH Stanford MS Barratt ES. Factor structure of the barratt impulsiveness scale. *J Clin Psychol.* (1995) 51:768–74. 10.1002/1097-4679(199511)51:63.0.co;2-1 8778124

[40] Xia C Wang D He Y. Study of self-rating idea of undergraduates in the mountain area of southern Zhejiang. *Chin J Sch Health.* (2012) 33:144–6. 10.16835/j.cnki.1000-9817.2012.02.006

[41] Turecki G Brent DA. Suicide and suicidal behaviour. *Lancet.* (2016) 387:1227–39. 10.1016/s0140-6736(15)00234-2 26385066 PMC5319859

[42] Lengua LJ Sandler IN West SG Wolchik SA Curran PJ. Emotionality and self-regulation, threat appraisal, and coping in children of divorce. *Dev Psychopathol.* (1999) 11:15–37. 10.1017/s0954579499001935 10208354 PMC8172092

[43] Maier MJ Schiel JE Rosenbaum D Hautzinger M Fallgatter AJ Ehlis AC. To regulate or not to regulate: emotion regulation in participants with low and high impulsivity. *Front Behav Neurosci.* (2021) 15:645052. 10.3389/fnbeh.2021.645052 34393732 PMC8363082

[44] Ochsner KN Gross JJ. The cognitive control of emotion. *Trends Cogn Sci.* (2005) 9:242–9. 10.1016/j.tics.2005.03.010 15866151

[45] Weger M Sandi C. High anxiety trait: a vulnerable phenotype for stress-induced depression. *Neurosci Biobehav Rev.* (2018) 87:27–37. 10.1016/j.neubiorev.2018.01.012 29407523

[46] Wardenaar KJ van Veen T Giltay EJ de Beurs E Penninx BW Zitman FG. Development and validation of a 30-item short adaptation of the mood and anxiety symptoms questionnaire (MASQ). *Psychiatry Res.* (2010) 179:101–6. 10.1016/j.psychres.2009.03.005 20472297

[47] Helmreich I Wagner S Mergl R Allgaier AK Hautzinger M Henkel V Sensitivity to changes during antidepressant treatment: a comparison of unidimensional subscales of the inventory of depressive symptomatology (IDS-C) and the hamilton depression rating scale (HAMD) in patients with mild major, minor or subsyndromal depression. *Eur Arch Psychiatry Clin Neurosci.* (2012) 262:291–304. 10.1007/s00406-011-0263-x 21959915

[48] DeCou CR Comtois KA Landes SJ. Dialectical behavior therapy is effective for the treatment of suicidal behavior: a meta-analysis. *Behav Ther.* (2019) 50:60–72. 10.1016/j.beth.2018.03.009 30661567

[49] Norr AM Allan NP Reger GM Schmidt NB. Exploring the pathway from anxiety sensitivity intervention to suicide risk reduction: chained mediation through anxiety and depressive symptoms. *J Affect Disord.* (2018) 231:27–31. 10.1016/j.jad.2018.01.015 29426035

[50] Johnson SL Zisser MR Sandel DB Swerdlow BA Carver CS Sanchez AH Development of a brief online intervention to address aggression in the context of emotion-related impulsivity: evidence from a wait-list controlled trial. *Behav Res Ther.* (2020) 134:103708. 10.1016/j.brat.2020.103708 32896743

[51] Lockwood J Daley D Townsend E Sayal K. Impulsivity and self-harm in adolescence: a systematic review. *Eur Child Adolesc Psychiatry.* (2017) 26:387–402. 10.1007/s00787-016-0915-5 27815757 PMC5364241

